# Long-Range Control of Class Switch Recombination by Transcriptional Regulatory Elements

**DOI:** 10.3389/fimmu.2021.738216

**Published:** 2021-09-14

**Authors:** Audrey Dauba, Ahmed Amine Khamlichi

**Affiliations:** Institut de Pharmacologie et de Biologie Structurale, IPBS, Université de Toulouse, CNRS, Université Paul Sabatier, Toulouse, France

**Keywords:** *IgH* locus, class switch recombination, switch transcription, enhancer, insulator, long-range interactions, chromatin loop extrusion

## Abstract

Immunoglobulin class switch recombination (CSR) plays a crucial role in adaptive immune responses through a change of the effector functions of antibodies and is triggered by T-cell-dependent as well as T-cell-independent antigens. Signals generated following encounter with each type of antigen direct CSR to different isotypes. At the genomic level, CSR occurs between highly repetitive switch sequences located upstream of the constant gene exons of the immunoglobulin heavy chain locus. Transcription of switch sequences is mandatory for CSR and is induced in a stimulation-dependent manner. Switch transcription takes place within dynamic chromatin domains and is regulated by long-range regulatory elements which promote alignment of partner switch regions in CSR centers. Here, we review recent work and models that account for the function of long-range transcriptional regulatory elements and the chromatin-based mechanisms involved in the control of CSR.

## 1 Outline of CSR In and Out of Germinal Centers

B lymphocytes have a remarkable ability to somatically alter their immunoglobulin (*Ig*) loci at different stages of their development. In developing B cells, *Ig* loci undergo V(D)J recombination catalyzed by the RAG1/RAG2 (RAG) complex. V(D)J recombination targets the variable regions of both Ig heavy chain (*IgH*) and Ig light chain (*IgL*) loci and lies at the basis of the vast primary antibody repertoire (1–4). Upon antigen challenge, mature B cells can further diversify the variable regions of *IgH* and *IgL* genes through somatic hypermutation (SHM) and the constant (*C_H_*) genes of the *IgH* locus through class switch recombination (CSR). The enzyme activation-induced cytidine deaminase (AID) is absolutely required for SHM and CSR and initiates these processes *via* transcription-dependent cytosine deamination of single-stranded DNA targets (5–9).

Depending on the type of the eliciting antigen, humoral responses are classically categorized in T-cell-dependent and T-cell-independent responses. SHM is a hallmark of affinity maturation featuring an increase in the affinity of antibodies (Abs), as an outcome of SHM in germinal centers (GCs) in the context of T-cell-dependent responses (5, 10). In a typical GC response, SHM generates a pool of mutated B cells that compete for a variety of signals required for their survival, delivered by the other GC-resident cells in an affinity-dependent manner. Positively selected B cells, with higher-affinity B-cell receptors, ultimately produce memory B cells and long-lived Ab secreting plasma cells, which provide effective protection against future reinfection (10).

CSR occurs *in vivo* following immunization or infection and enables antigen-activated, IgM^+^-expressing B cells to change the constant domains of Igµ heavy chains, hence the expression of novel isotypes (IgG, IgE, or IgA) with different effector functions (11–13). Switching from IgM to other isotypes depends on the nature of antigen, the cytokines produced by other immune cell types, and the interactions engaging activated B cells with the other immune cell types (helper T cells, dendritic cells…) (11–13). The signals received by the B cell trigger different signaling pathways that induce a complex interplay between 3D conformational changes of the *IgH* locus, epigenetic modifications, and transcriptional programs that mobilize a set of transcription factors that induce or suppress transcription of *C_H_* genes (6, 8, 14–17).

Besides CSR induced in T-cell-independent responses which do not involve GC formation, CSR in the context of T-cell-dependent responses has long been assimilated to GCs (10, 18). However, seminal observations on the kinetics of switch transcripts appearance and CSR [e.g., (19–21)] suggested that CSR occurs outside GCs. This notion recently gained support from the analyses of the earliest stages of an immune response, showing CSR at the early onset of GC formation, prior to SHM (22).

CSR is usually triggered *in vitro* by culturing splenic B cells in the presence of various cocktails of cytokines and/or mitogens which induce both AID and CSR. For instance, mouse B cells are typically induced to switch to IgG3 and IgG2b when activated with lipopolysaccharide (LPS) and to IgG1 and IgE in the presence of LPS+IL4 or anti-CD40+IL4. These culture systems allow the investigators to address B-cell-autonomous mechanisms that are more difficult to tackle in the context of the complex molecular processes and cellular interactions triggered *in vivo* by antigens (6).

Most, if not all, of our knowledge on the transcriptional elements that control CSR derives from the use of cultured splenic B2 B cells, the main B-cell population in the spleen. However, CSR can also take place in B1 B cells, which form the major population in the pleural and peritoneal cavities. B1 B cells have a distinct antigen specificity, display different cell surface markers, and switch to IgA preferentially (23, 24). However, the transcriptional mechanisms involved in CSR in B1 B cells have just begun to be investigated.

CSR is not restricted to activated mature B cells. It has long been known that it can occur in developing B cells, though at a low frequency. Indeed, various studies described CSR events in Abelson murine leukemia virus (A-MuLV)-transformed pro-B lines [e.g., (25–29)] and early primary B cells as well [e.g., (30–35)]. In fact, seminal discoveries on the importance of transcriptional mechanisms in CSR were made by using pro-B and pre-B lines [e.g., (36–38)]. Nonetheless, here too, there is still much to learn about the transcriptional elements that control CSR.

**Figure 1:**
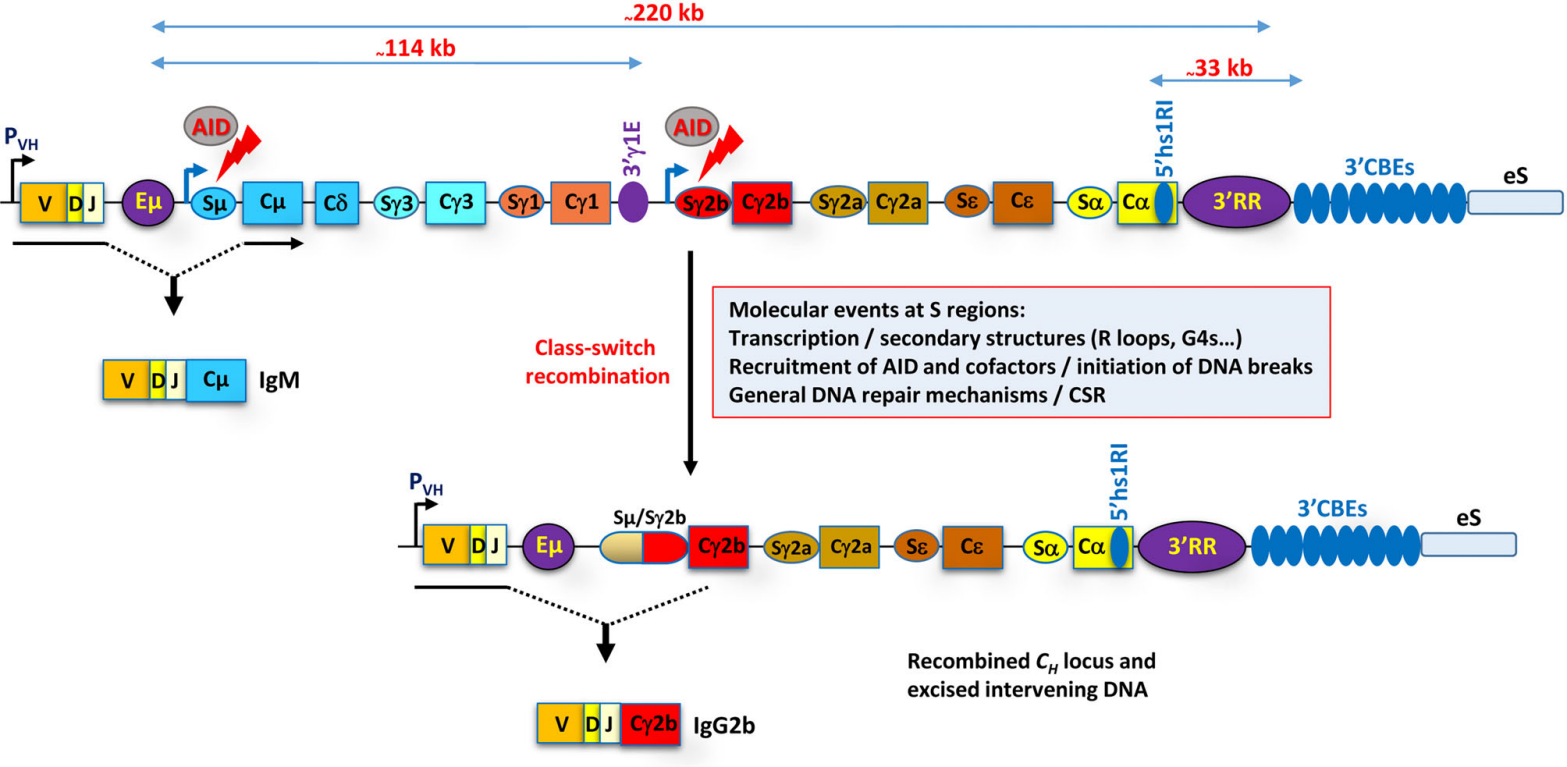
Rearranged mouse *IgH* locus. The various regulatory elements, Eµ, 3'γ1E, 5'hs1RI, and 3'RR, and the 3' CTCF binding elements (3'CBEs) are depicted. Approximate distances are indicated on the top of the scheme. The promoter of the rearranged V(D)J gene is indicated by a black arrow. With the exception of *Cδ*, the *C_H_* genes are structurally similar. They are composed of an I promoter followed by an I exon; highly repetitive, GC-rich S regions; and C_H_ exons (not depicted). The core S sequences vary in size, the shortest being Sε (_~_1 kb) and the largest Sγ1 (_~_10 kb), and contain characteristic repeated motifs including AID target motifs. The Iµ promoter is constitutive and coincides with the core Eµ enhancer, while the other I promoters are signal dependent and have typically no enhancer function. The constitutive Iµ promoter and (in this example) the induced Iγ2b promoter drive the transcription of Sµ and Sγ2b, respectively (blue arrows). AID targets the transcribed Sµ and Sγ2b regions (red arrows) and initiates DSBs. Repair of the breaks ultimately leads to CSR (fused Sµ/Sγ2b oval). Consequently, the IgM^+^-expressing B cell switches to the expression of IgG2b (in this example) with novel effector functions. The eS region downstream of the 3'CBEs stands for ectopic S-like region (see main text for details and the table associated with **Figure 2**).

Regardless of the developmental stage, CSR occurs between highly repetitive switch (S) sequences, located upstream of the *C_H_* gene exons, whose transcription is mandatory for CSR, and is driven by specific promoters (called I promoters) in a signal-dependent manner (6, 8, 14) (**Figure 1**). Switch transcription (ST) targets AID activity, which initiates DNA cleavage by deaminating exposed cytosines into uracils at the universal donor, Sμ region, and the activated downstream S region. The uracils are processed by the base excision and mismatch repair pathways, ultimately leading to double-strand break (DSB) intermediates. The DSBs are taken in charge by the DNA damage response pathway and repaired by the classical and alternative non-homologous end joining pathways (9, 39, 40).

ST is controlled by various distant *cis*-acting elements, described in detail below. This control often involves long-range interactions that juxtapose transcribed partner S sequences and promote CSR initiation. In this review, we mainly summarize recent work and models on the activity of these regulatory elements and on the long-range chromatin-based mechanisms that control ST and CSR.

## 2 *IgH* Transcriptional Elements That Control CSR

The critical transcriptional elements involved in ST and CSR have long been thought to be confined within the *C_H_* region, bordered by the Eµ enhancer and the 3' CTCF binding elements (3'CBEs) (**Figure 1**). However, recent studies involved additional remote non-*IgH* elements in the control of CSR. Here, we will focus on the role of enhancers and CTCF insulators as revealed by mutational studies on the endogenous murine *IgH* locus.

### 2.1 The Lingering Mystery of Eµ Enhancer

**Figure 2:**
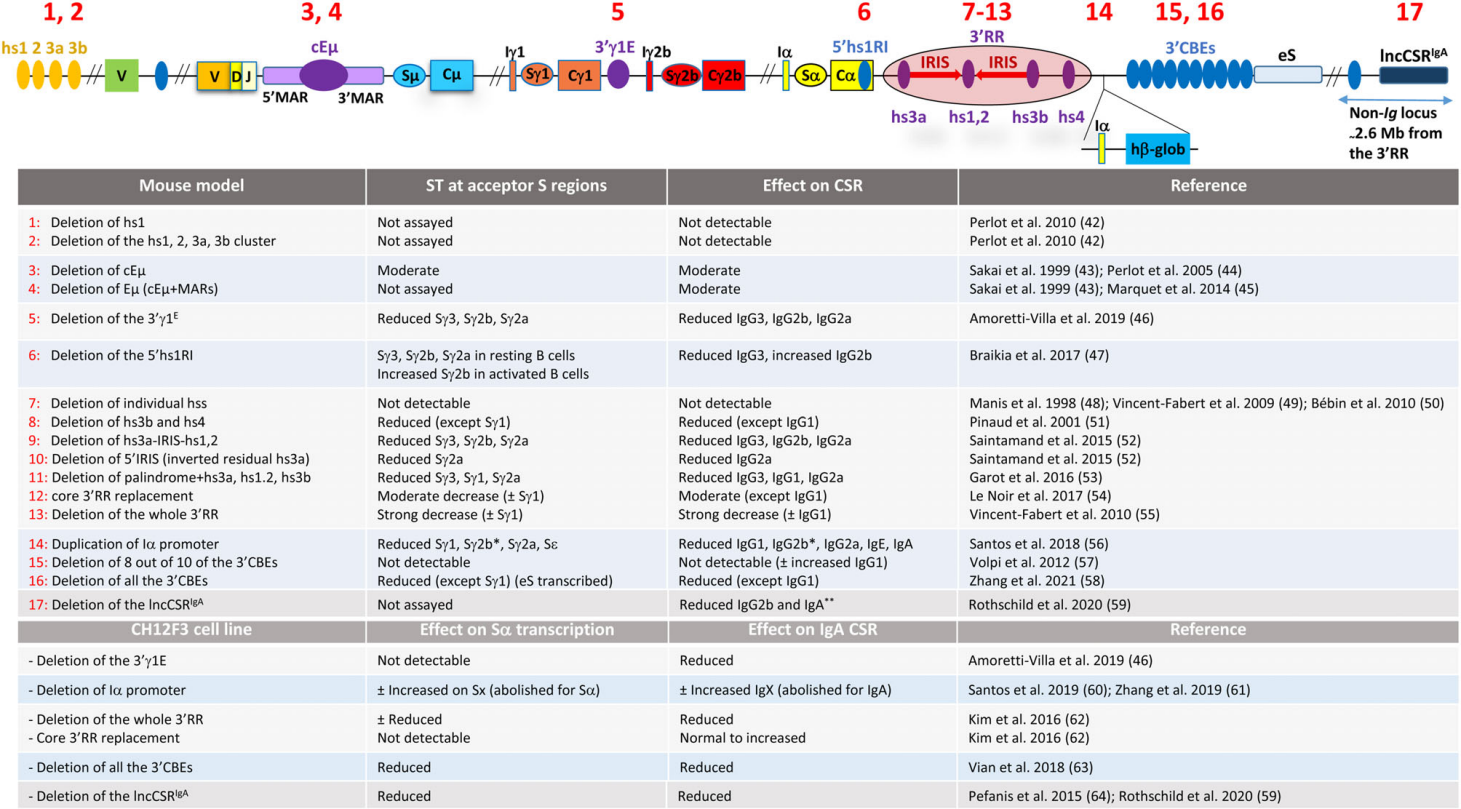
Effects of the mutations of the *IgH* and non-*Ig* regulatory elements on ST and CSR. The various mutations in mice and in CH12 cell line are numbered in the upper scheme. At the 5' part of the locus, hs1, hs2, hs3a, and hs3b are a cluster of four DNase I hypersensitive sites located some 30 kb upstream of the most distal V_H_ gene segment. hs1 is pro-B specific, binds various transcription factors, and exhibits a moderate repressive transcriptional activity as detected in transient transfection assays. The core enhancer Eµ (cEµ) and the flanking matrix attachment regions (MARs) are depicted. Within the 3'RR, the hs1,2 enhancer lies at the center of a large palindrome. The palindrome is bordered by inverted copies of hs3 enhancer. hs4, in contrast, lies outside the palindrome. hβ-glob stands for the human β-globin exon whose transcription is driven by the mouse Iα promoter (see main text for details). The table is a summary of the targeted mutations outlined in the upper scheme and their effect. ± indicates low to moderate. The asterisk on γ2b in mutation 14 means that Sγ2b transcription and IgG2b CSR are reduced following LPS stimulation but are normal upon TGF-β stimulation. The two asterisks on IgA in mutation 17 indicate that CSR to IgG2b and IgA are reduced in activated splenic B cells, while in Peyer’s patch B cells, only IgA CSR is reduced. Sx and IgX stand for Sγ3, Sγ1, Sγ2b, Sγ2a, Sε, and the corresponding isotypes, respectively, and eS stands for ectopic S-like region [updated from (17)].

The Eµ enhancer comprises the core enhancer (cEµ) flanked by matrix attachment regions (41) (**Figure 2**). The cEµ coincides with Iµ promoter, which likely explains the constitutive transcriptional activity of Iµ (65, 66), contrasting in this regard with the inducible activity of downstream I promoters.

Deletion of Eµ led to a dramatic decrease of IgM^+^ population in Peyer’s patches but did not affect the number of B cells engaged in the GC reaction (45). In the spleen, the number of follicular (FO) B cells was significantly reduced, whereas the number of marginal zone (MZ) B cells was unaffected. Nonetheless, surface staining revealed that MZ and FO B cells expressed comparable levels of IgM, suggesting that Eµ deletion did not impact µ heavy chain (HC) gene expression in mature B cells (45). Slightly reduced IgG1 serum levels were found in Eµ-deleted mice, which otherwise exhibited normal response upon immunization (45).

The role of Eµ enhancer in CSR is far from clear since deletion of either cEµ or Eµ enhancer only marginally affected CSR (43–45). In particular, cEµ deletion markedly reduced Iµ transcript levels (44) but had no apparent effect on ST of acceptor S regions (67), on surface Ig expression or *IgH* isotype serum levels (43, 44) (**Figure 2** and associated table). The moderate effect of Eµ enhancer on CSR is surprising as Eµ activates the universal donor Sµ and interactions with the 3'RR and other essential elements for CSR (see below), suggesting the presence of redundant elements that render Eµ enhancer dispensable in activated mature B cells.

### 2.2 The *IgH* Locus Got Its Super-Enhancer: The 3' Regulatory Region

The major *IgH* control element in mature B cells is a long-range super-enhancer termed 3' regulatory region (3'RR) (17, 68) (**Figure 2**). The 3'RR (_~_28 kb) is composed of four B-cell-specific enhancers, hs3a, hs1,2, hs3b, and hs4, that act in synergy. hs1,2 is flanked by inverted repeated intervening sequences (IRISs) and lies at the center of a large palindromic region bordered by two inverted copies of hs3, hs3a and hs3b, whereas the distal hs4 enhancer is located outside of the palindrome (17, 68).

Beyond its key role in CSR discussed below, the 3'RR was also shown to control SHM (69) and *IgH* expression (51, 55), thus revealing the centrality of the 3'RR in the major molecular processes that take place at the *IgH* locus in activated mature B cells and plasma cells.

Deletion of the 3'RR markedly reduced the number of MZ B cells with no obvious effect on FO B cells. Nonetheless, the deletion impacted surface IgM expression on both populations (70). Among the 3'RR enhancers, hs4 appears to maintain µ gene expression in unstimulated MZ and FO B cells. However, upon antigen activation, hs4 is no longer required for this maintenance (53). Instead, the upstream 3'RR enhancers jointly gain a prominent role and control SHM, CSR, and Ig production (53).

Deletion of individual 3'RR enhancers had no effect on B-cell proliferation, ST, CSR, Ig serum isotype production, percentage of FO and MZ B cells in the spleen, or antigen-specific responses, suggesting a redundancy between these elements (48–50). In contrast, joint deletion of hs3b/hs4 severely impaired ST and CSR to all isotypes except for IgG1, which was only reduced (51). When the whole 3'RR was deleted, ST of and CSR to all isotypes were inhibited, with notable exception of Sγ1 ST and IgG1 CSR, which were severely reduced but readily detectable. Sµ transcript levels were also reduced in 3'RR-deleted B cells, though the reduction was moderate compared with downstream switch regions (55) (**Figure 2**).

An important question concerning the function of the 3'RR relates to the relative contribution of the core enhancers *versus* the whole structure of the 3'RR, in particular its large palindrome. In this regard, removal of the proximal hs3a-left IRIS-hs1,2 region reduced Sγ3, Sγ2b, and Sγ2a transcription (**Figure 2**). Ig production of all isotypes was significantly reduced *in vitro*, while only IgG3 and IgG2a serum levels were reduced (17, 52). When the left IRIS alone was deleted, but leaving intact hs3a and hs1,2 enhancers, only Sγ2a transcription and IgG2a surface expression were reduced, while IgG3 and IgG2a serum titers were reduced (52). Overall, when the large proximal deletion encompasses hs3a and hs1,2, there is a strong reduction of ST and CSR to a subset of S regions, while deletion of the IRIS alone preferentially targets Sγ2a.

When the entire palindrome (including hs3a, hs1,2 and hs3b) was deleted, Sγ3 and, to a lesser extent, Sγ1 and Sγ2a transcription, and CSR to the corresponding isotypes were impaired (53) (**Figure 2**). Interestingly, replacement of the whole endogenous 3'RR by the four core enhancers led to an overall moderate defect in ST of all isotypes (54) (**Figure 2**). Thus, the palindrome appears to be required for efficient ST and CSR.

In another mouse line, Iα promoter was inserted downstream of the 3'RR (47), preserving the integrity of the 3'RR (**Figure 2**). Of the ectopic and the endogenous Iα promoters, only the ectopic promoter was active in resting B cells. Following stimulation, the ectopic Iα was further induced, together with the endogenous Iα (47, 56). The duplication reduced Sγ1, Sγ2a, and Sε transcription and CSR to the corresponding isotypes. Surprisingly, IgA CSR was reduced despite apparently normal Sα transcript levels. The pattern of Sγ2b activation depended on the type of stimulation. LPS stimulation reduced Sγ2b transcripts and IgG2b CSR levels. In contrast, TGF-β stimulation (which also activates Iα) led to normal Sγ2b transcripts and IgG2b CSR levels (56).

Nonetheless, as discussed (17), a potential caveat in these studies relates to 3'RR transcription and associated enhancer RNAs (eRNAs) which correlate with its activity (71–73). It is still unknown whether and how a large deletion of an IRIS or a close alignment of the core enhancers affects 3'RR eRNA structure, stability, and function (see below). It is possible that the effect on ST and CSR results from missing or destabilized eRNAs rather than from the absence of an IRIS per se. Similarly, whether the active ectopic Iα promoter perturbs the architecture of the 3'RR or interferes with transcription elongation within or downstream of the 3'RR remains to be investigated (17).

In conclusion, the whole 3'RR is the master element in the control of ST, CSR, SHM, and *IgH* expression. The 3'RR controls CSR by regulating ST, but this correlation is not absolute. Components of the 3'RR may display some isotype preference. Overall, the 3'RR only moderately impacts ST at Sµ region. The 3'RR core enhancers display redundancy but act in synergy for efficient CSR, and the global structure of the 3'RR seems to contribute to its full activity (17).

#### 2.2.1 When the *IgH* Locus Starts to Transvect: The 3'RR and Inter-Allelic Recombination

Most of the mutational studies conducted on the endogenous *IgH* locus concluded to a *cis*-regulation of ST and CSR by the 3'RR through a long-range effect on I promoters (17). However, the possibility remained that inter-allelic recombination could contribute to CSR. The bi-allelic nature of ST (74–76), the long-known frequent occurrence of CSR on both chromosomes [e.g., (77, 78)] and the recurrent involvement of switch regions in chromosomal translocations (79), made such scenario plausible. Besides the peculiar case of rabbit, featuring 13 *Cα* genes (80, 81), detection of presumably infrequent inter-allelic switch recombination at the endogenous *IgH* locus of other species required special genetic tools.

In a mouse model in which one *IgH* allele was engineered so that VDJ-Cµ transcription was suppressed (and *trans*-splicing prevented), sequencing of cDNAs revealed that inter-allelic recombination accounted for up to 7% of recombination events to *Cα* in Peyer’s patches and up to 13% to *Cγ3* in LPS-activated splenic B cells (82). Upon crossing with mice devoid of hs3b/hs4, hence deficient in CSR (51), and sequencing of switch junctions in activated hemizygous B cells, it was found that the CSR-deficient allele (with deleted hs3b/hs4) could complement the excluded allele (with suppressed VDJ-Cµ transcription) through inter-allelic recombination (83). Another mouse model bearing a wild-type allele and a 3'RR-deficient allele enabled the same group to tackle directly the *trans*-effect of the 3'RR. It was found that the 3'RR of the wild-type allele could promote SHM and CSR on the second, 3'RR-deficient allele (on which both SHM and CSR are deficient) (84).

Thus, in addition to its established role as a major *cis*-regulatory element of SHM and CSR, the 3'RR can also operate in *trans* to control these processes in a fraction of activated B cells.

#### 2.2.2 When B Cells Become Suicidal: The 3'RR and Locus Suicide Recombination

The observation that the 3'RR was highly enriched in switch-like repeats (85) and that it was transcribed upon activation of mature B cells for CSR (73) raised the possibility that the 3'RR could be the target of a CSR-like process (17). Unlike classical CSR, however, recombination between Sµ and the 3'RR would delete the whole *C_H_* region and part of or the whole 3'RR (73), leading to the loss of surface Ig expression required for B-cell survival. It was thus proposed that this CSR-like process was important for B effector cell differentiation and homeostasis, for instance by counterselecting activated mature B cells with harmful Ig specificities (73). This phenomenon, termed locus suicide recombination (LSR) (73), was reported in both mice and humans and was AID dependent (73, 86). The binding profiles of AID and RNA polymerase II (RNAPII) at the 3'RR and flanking sequences were similar (73, 87). LSR was initially reported to occur at levels approaching classical CSR by PCR/Southern blot on excised episomal circles (73), though not by more sensitive techniques (58, 88, 89).

It is presently unclear if LSR is an active and autonomous process driven by specific mechanisms that co-opt classical CSR. Alternatively, LSR could be a by-product of bona fide CSR, resulting from an accidental attack of the transcribed 3'RR by AID. Further studies are required to elucidate the mechanisms that underlie LSR and its physiological significance.

#### 2.2.3 The 3'RR and the Curious Case of IgD

It has long been established that IgD was co-expressed with IgM on the surface of naive mature B cells and that δ HC production resulted from alternative splicing of a long primary transcript encompassing Cµ and Cδ exons (90, 91). IgD CSR is a rare event and was mostly studied in humans in whom IgD CSR is relatively abundant in B cells that populate the upper aerodigestive mucosa-associated lymphoid tissues (91). The *Cδ* gene is unique in that it has no canonical switch sequence. Nonetheless, the gene has a switch-like sequence termed σδ, upstream of Cδ exons, that can recombine with Sµ (91). IgD CSR is rare in mouse and is not detectable in splenic B cells but was readily detected in mouse mesenteric lymph nodes (92). Surprisingly, IgD CSR was found to be 3'RR independent (92), contrasting in this regard with CSR to other isotypes. The transcriptional elements that control CSR to IgD remain to be identified.

#### 2.2.4 The 3'RR and CSR in B1 B Cells: It May Depend on Which B Cell You Are

A plethora of mutational studies established the central role of the 3'RR in activated B2 B cells with the unspoken assumption that this role extended to the B1 B cells as well. However, in contrast to B2 B cells, IgA CSR in activated B1 B cells was reported to be 3′RR independent (93). Surface expression of IgA was normal in *in vitro*-activated 3'RR-deficient B1 B cells, but IgA titers were markedly reduced in culture supernatants, and this correlated with decreased Iµ-Cα post-switch transcript levels (93). Nonetheless, it is unclear if Sα pre-switch transcription was affected. Thus, it was proposed that though dispensable for IgA CSR in B1 B cells, the 3'RR was required for efficient transcription of the switched *Cα* gene (93).

### 2.3 The 3'γ1E Enhancer: Better Few Constant Genes Than Nothing

Previous 4C-Seq analyses identified a PAX5-dependent hs site downstream of *Cγ1* gene (hereafter 3'γ1E) that bound multiple transcription factors in *Rag2*-deficient pro-B cells (94). In particular, the 3'γ1E exhibited a pro-B-cell-specific enhancer activity (95) and bound the MED1 subunit of the Mediator complex (95, 96).

In activated mature B cells, the 3'γ1E also bound MED1 and MED12 subunits of the Mediator complex and was transcribed (97). 4C-Seq experiments revealed that the 3'γ1E interacted with Eµ and the 3'RR (97). In 3'γ1E-deficient mice, activated B cells displayed defective ST across Sγ3, Sγ2b, and Sγ2a and CSR to the corresponding isotypes (**Figure 2**) (46).

Thus, the 3'γ1E emerges as a novel element that regulates CSR in an isotype-specific manner (46), adding an additional layer of complexity to the long-range mechanisms that operate at the *IgH* constant locus.

### 2.4 CTCF Binding Elements: Guardians of the Temple and Insiders

CTCF is a multivalent 11 zinc finger (ZF) protein thought to bind uncommonly long and diverse DNA sequences through different combinations of its 11 ZFs (98). These combinations are not arbitrary. Extensive mutational and ChIP-Seq analyses of _~_50,000 genomic sites in primary B lymphocytes found that CTCF reads sequence diversity through ZF clustering by grouping contiguous ZFs into distinct binding subdomains (99). Broadly outlined, the central ZFs 4–7 were found to anchor CTCF to _~_80% of CBEs containing the core motif. Peripheral ZFs associate with non-conserved flanking DNA sequences as functional clusters and modulate CTCF binding *in vivo* (99). CTCF was involved in various processes ranging from transcriptional regulation and insulator activity to chromatin boundary formation (17, 100). Its role in chromatin loop formation during CSR is discussed below.

The role of CTCF in CSR was investigated through a conditional knockout of the mouse *Ctcf* gene (101). Interestingly, CTCF loss led to increased transcript levels of Sγ3, Sγ1, and Sγ2b in unstimulated but not in activated splenic B cells, associated with an apparently increased CSR to IgG3, IgG1, and IgG2b. In contrast, CTCF depletion had no significant effect on Sµ transcription or AID expression (101).

These findings strongly suggest that CTCF acts, at least in part, by preventing premature activation of I promoters (101).

#### 2.4.1 The 5'hs1RI Insulator

A hs was identified within the last intron of the *Cα* gene (102), which binds CTCF and cohesin in resting B cells (103), but evicts CTCF though not cohesin upon activation (47, 101, 103). This element, termed 5'hs1RI (**Figure 1**), is conserved in the human *Cα1* and *Cα2* genes (17, 47).

In 5'hs1RI-deleted mice, Sγ3 and, to a lesser extent, Sγ2b and Sγ2a transcripts were specifically upregulated in unstimulated splenic B cells (47). In activated B cells, increased CSR to IgG2b correlated with increased Sγ2b transcription; however, CSR to IgG3 were defective despite abundant Sγ3 transcripts. It is still unclear whether this is due to promoter interference or to other mechanisms (47). Notwithstanding, the data strongly suggest that 5′hs1RI is involved in the transcriptional silencing of Iγ3, Iγ2b, and Iγ2a, but not of Iγ1, Iε, and Iα promoters.

Overall, the 5'hs1RI emerges as an inducible CTCF insulator that regulates the temporal expression of a subset of *C_H_* genes, by blocking premature activation of their promoters prior to B-cell activation (47).

#### 2.4.2 The *IgH* Super-Anchor: 3'CTCF Binding Elements

Multiple hs elements were identified downstream of hs4 enhancer, some of them exhibiting insulator activity *in vitro* (104). This region, also termed super-anchor, consists of 10 CBEs (57, 105). Deletion of the first eight CBEs in mice had at best a modest increase of CSR to IgG1 (63) (**Figure 2**), and the crosslinking frequencies (by 3C assays) of the 3'RR with Eµ or with I promoter regions were not altered (63). However, the fact that the deletion spared two CBEs prevented a definitive conclusion on the role of the super-anchor in CSR and the architecture of the locus.

This issue was solved in two systems. Deletion of the 10 3′CBEs in CH12F3 B lymphoma cell line led to _~_2-fold decrease of CSR to IgA and a moderate decrease of Sα transcript levels, suggesting a role for the 3'CBEs in 3'RR/Iα promoter interactions (106). Nonetheless, because activated CH12 cells switch exclusively to IgA (upon stimulation with TGFβ-containing cocktails), the impact of the 3'CBEs on the other isotypes remained unclear.

In this regard, deletion of the whole 3'CBEs cluster was recently performed in chimeric mice generated by RAG2-deficient blastocyst complementation (58) and CSR assayed by CSR-HTGTS. Except for Sγ1 transcripts and CSR to IgG1 whose levels were unaffected, CSR to all other isotypes was reduced, and this correlated with varying degrees of reduced ST of the corresponding S regions (58).

Together, the data from CH12 cells (106) and chimeric mice (58) revealed that the 3'CBEs promote ST of and CSR to all downstream S regions with the exception of Sγ1.

Interestingly, GRO-Seq analysis revealed that, upon deletion of the 3'CBEs, the 30-kb region just downstream [termed ectopic S (eS) region] (**Figure 1**) becomes transcriptionally active in both sense and antisense orientations in unstimulated splenic B cells (58). Following activation, the eS region is further transcribed, generating convergent transcription that may facilitate AID recruitment. 3C-HTGTS data showed that the eS region interacts with the Eµ–Sµ region, suggesting a synapsis between Sµ and eS regions (58). Accordingly, CSR-like junctions involving Sµ and sequences within the first 6 kb of the eS region were detected and accounted for 1%–3% of all CSR-related junctions (58).

Thus, the 3'CBEs act as an insulator that prevents transcriptional activation of the eS region and its recombination with Sµ region during CSR.

## 3 Signals and Regulatory Elements That Control Switch Recombination in Developing B Cells

Various studies involved signaling through Toll-like receptors in the induction of AID expression and CSR in early B cells [e.g., (31, 32, 107)]. Recently, interleukin 7 (IL7) was involved in the control of ST by repressing Iγ3 and, to a lesser extent, Iγ2b promoter in cultured wild-type pro-B cells (108). Nonetheless, LPS stimulation induced Sγ3 and Sγ2b transcription and CSR to Sγ3 and Sγ2b, respectively (108). Sγ1 and Sε transcript levels, though undetectable in cultured pro-B cells, were also increased following LPS+IL4 stimulation (108).

With regard to transcriptional elements, the 5'hs1RI suppressed Sγ3 and, to a lesser extent, Sγ2b transcription in unstimulated pro-B and pre-B cells (47). Interestingly, removal of 5'hs1RI led to increased levels of Sγ3 and Sγ2b transcripts in the absence of detectable 3'RR eRNAs (47). Along similar lines, duplication of Iα promoter downstream of the 3'RR led to a premature activation of the ectopic Iα at the pro-B-cell stage, while the endogenous Iα promoter remained silent (47). These observations indicate that the 3′RR activity at the pro-B-cell stage does not require 3′RR transcription (i.e., 3'RR eRNAs) (47). Together, the above findings strongly suggest that IL7/IL7R pathways and the 5'hs1RI are part of active processes that operate in developing B cells to keep in check, through yet unknown mechanisms, ST and CSR (108).

## 4 Long-Range Regulation by *IgH* Control Elements: The Problem Is Not the Distance

### 4.1 Compete or Not Compete for the Control of CSR

An important question in the field of transcriptional regulation is whether promoters compete for, or are co-regulated by, a shared (and often distant) regulatory element. In the specific case of the 3'RR, it was known that activation of primary B-cell populations often induces more than one I promoter, the prevailing interpretation being that I promoters compete for 3′RR activity [e.g., (47, 48, 109–111)]. However, whether competition applied to I promoters located on the same chromosome and that responded to the same stimulus remained uncertain. The issue was complicated by the finding that ST can occur on both alleles (74–76), so that even the use of single cells does not settle this issue.

The use of mouse models with engineered endogenous *IgH* locus, polymorphic allelic differences, and a single allele-specific RT-qPCR assay revealed that the type of stimulation largely determined which mode of *cis*-activation, competition or co-activation, prevailed (112). In the presence of IL4, the majority of alleles displayed promoter competition, but Sγ1 single expressers prevailed over Sε single expressers. In the presence of TGF-β, there was also competition between Iγ2b and Iα, but the percentages of single Sγ2b- and Sα-expressing alleles were similar (112). In contrast, Iγ3 and Iγ2b promoters were co-activated upon LPS stimulation. Moroever, Iγ2b promoter was often activated on alleles with pre-activated Iγ3. These findings strongly suggest that 3′RR activity, RNAPII, and transcription factors and co-factors are not limiting during I promoter activation and that initial activation of one promoter does not prevent activation of the other (17, 112). In particular, the Iγ2b promoter, which is induced by both LPS and TGF-β, was co-activated with Iγ3 in the vast majority of alleles upon LPS stimulation, but was almost never co-activated with Iα after TGF-β stimulation (112). It was speculated that co-activation and competition reflect two kinetics of the activation of I promoters: co-activation of Iγ3 and Iγ2b promoters in the rapidly responding MZ B cells during T-independent responses and competition between the other I promoter pairs in FO B cells during the relatively delayed T-dependent responses (112).

The single-chromosome approach also solved the long-standing issue of the polarity of the 3'RR, i.e., if the 3'RR activity was exclusively oriented toward the upstream I promoters or if it could also target a downstream promoter (17). In this regard, analysis at the single-chromosome level of activated B cells with duplicated Iα promoter downstream of the 3'RR (47) (**Figure 2**) revealed that the 3′RR activated both the ectopic and the endogenous Iα promoters, which points to a bidirectional activity (112).

The above studies revealed that the 3’RR has a bi-directional activity, and that the type of stimulation largely determines which mode of cis-activation, competition or co-activation, prevails.

### 4.2 Transcriptional and Epigenetic Regulation by the 3'RR

Mammalian genomes are predominantly methylated at cytosines in CpG dinucleotides. In general, unmethylated CpGs are associated with active promoters, while methylated CpGs are closely associated with transcriptionally silent promoters (113). The methylation patterns of various *cis*-acting elements at the *IgH* constant region were determined in primary B cells by bisulfite sequencing. Unexpectedly, the methylation profiles of almost all the *cis*-acting elements were established and faithfully maintained independently of B-cell activation or ST (114). The unmethylated pattern of Eµ and 3'γ1E and the hypermethylated pattern of 5'hs1RI did not change following B-cell activation or insulation of the 3'RR. Surprisingly, induction of ST did not impact the methylation profiles of I promoters: Iγ3 and Iγ2b were unmethylated in resting as well as in LPS-activated splenic B cells, while the hypermethylated profile of Iε for instance did not vary upon activation. The only exception was Iγ1 whose demethylation was induced. Importantly, the 3'RR-dependent Iγ3 and Iγ2b promoters remained unmethylated following insulation of the 3'RR, which fully repressed the two promoters. This implies that the long-range activation of these promoters by the 3'RR involves mechanisms that do not rely on DNA methylation (114).

A remarkable aspect of transcription elongation across switch regions relates to the marked stalling of RNAPII (66, 115) and the peculiar pattern of chromatin activating modifications (115–117) at these regions. In particular, induced histone acetylation and H3K4me3 mark extended over the entire switch regions irrespective of their length and dropped at C_H_ exons (115, 117). In contrast, these patterns were observed in the constitutively transcribed Sµ region in resting B cells and did not vary upon activation (115, 117).

Catalysis of methylation marks on H3K4 is effected by PTIP (PAX interaction with transcription activation domain protein), a component of the mixed-lineage leukemia 3 (MLL3)/MLL4 complex (118). Activated PTIP-deficient B cells exhibited a defect in Sγ3, Sγ1 and Sγ2b and CSR to IgG3, IgG1, and IgG2b; the effect on Sγ1 transcription was milder, whereas Sε transcript and IgE CSR were unaffected (117, 119, 120). On the other hand, the chromatin profiles of Sµ and the 3'RR were essentially unaffected (117). It was proposed that PTIP promotes ST by bridging the 3'RR to I promoters, as 3'RR/I promoter interactions are disrupted in activated PTIP-deficient B cells (119).

Transcriptional and epigenetic analyses of mice devoid of the 3'RR revealed a dramatic decrease of transcription initiation along the downstream Ix–Sx–Cx regions, while the Iµ–Sµ–Cµ region was only minimally affected (121). Similarly, while the deposition of H3Ac and H3K4me3 marks was severely reduced along the downstream S regions, the Iµ–Sµ–Cµ region was essentially unaffected (121). This trend was not seen for H4Ac deposition which remained intact in activated 3'RR deficient (121).

Thus, the 3'RR is the central element in the control of ST initiation and histone modifications at acceptor S regions. Nonetheless, some epigenetic modifications, illustrated by H4Ac mark and DNA methylation, are 3'RR independent.

### 4.3 The Cohesin and the Mediator Complexes and Long-Range Interactions in CSR

It is now admitted that the chromatin interaction landscape plays an important role in the epigenetic control of gene expression. Interactions between enhancers and target promoters generally take place within submegabase-sized topologically associating domains (TADs), where these interactions occur at higher frequency than with elements of different TADs (122, 123). Chromatin interactions between boundary elements that bind CTCF (CBEs) and the Cohesin complex tether the bases of loops and separate the TADs from each other, thus preventing ectopic enhancer–promoter interactions (122–125). However, this is not an absolute rule as long-range interactions are not always blocked by CTCF and Cohesin binding to CBEs (126), and some of these sites can rather facilitate gene activation (122–124). Various studies revealed that juxtaposition of TAD boundaries by CTCF is strongly biased toward convergent CBEs (127–130). Within TADs, the Cohesin and the Mediator complexes are important for the formation of enhancer/promoter chromatin loops. Cohesin is loaded at these loops by the cohesin-loading factor NIPBL, which also binds the Mediator complex (131–133).

In pro-B cells, it was shown that the *IgH* locus spans a multi-megabase-sized TAD divided into three sub-TADs; one of these sub-TADs extends from the proximal V_H_ domain to the 3'CBEs (134). It is in that sub-TAD that most events pertinent to ST and CSR take place and, for the most parts, in the domain extending from the Eµ region to the 3'CBEs (**Figure 2**). In this chromatin domain, Eµ enhancer associates with the 3'RR in both unstimulated and activated B cells (67). Surprisingly, cEµ deletion only marginally impacted Eµ/3'RR association (67). In resting B cells, Eµ, the 3'RR, and I promoters, especially Iγ3, were poised for ST activation, and it was proposed that this poised configuration facilitates I promoter activation (67). Depending on the nature of stimulation, I promoters were recruited to the Eµ/3'RR complex leading to a juxtaposition of Sµ and the downstream switch partner (67).

Subsequent analyses by ChIP-Seq found that CTCF and Cohesin were recruited to the 3'CBEs in unstimulated B cells, with no significant enrichment at the Eµ region. Following stimulation, Cohesin was recruited to the Sµ–Cµ region, though not to Eµ, in a CTCF-independent manner (103). In the CH12 line, knockdown of SMC1 and SMC3 core subunits of the Cohesin complex or of NIPBL and WAPAL loader/unloader subunits reduced IgA CSR, a clear indication that the Cohesin complex was required for CSR (103).

The Mediator complex was also involved in ST and CSR. In unstimulated B cells, the MED1 and MED12 subunits were specifically recruited to Eµ enhancer and 3'RR (97). Following stimulation, the two subunits were recruited to Eµ, 3'RR, 3'γ1E, and the induced I promoter, in a stimulation-dependent manner (97). A conditional knockout of *Med1* led to reduced ST of all acceptor S regions and CSR to the corresponding isotypes in activated B cells. These findings strongly suggested that the Mediator complex promoted ST at downstream S regions (97). In agreement with previous findings on unstimulated B cells (67), 4C-Seq experiments detected strong interactions between Eµ and the 3'RR as well as a preferential association with the Iγ3 region (97). Upon stimulation, interactions between Eµ, 3'RR, 3'γ1E, and the activated I promoter were readily detected, and the pattern of these interactions correlated with MED1 and MED12 recruitment. Accordingly, Eµ/3'γ1E/I promoter interactions were reduced in MED1-depleted B cells (97). Altogether, these findings suggested that the Mediator and the Cohesin complexes promoted ST of downstream switch regions and were required for the long-range interactions between the *IgH* transcriptional *cis*-acting elements (97).

### 4.4 A Role for Non-Coding RNAs in the Long-Range Control of CSR

#### 4.4.1 Regulation of the Transcriptional Activity of the 3'RR

Enhancer transcripts (eRNAs) have (relatively) recently emerged as potentially essential for enhancer activity. These non-coding RNAs have been involved in the regulation of gene expression at different levels, for instance by stabilizing or trapping factors that bind enhancers, by generating and/or stabilizing chromatin loops that facilitate interactions between enhancer and target promoters, and by releasing paused RNAPII for productive transcriptional elongation (135). Yet, the mechanisms of action of eRNAs are still unclear. Moreover, whether it is the act of transcribing the enhancer or the eRNAs themselves that are crucial for enhancer activity has not been definitively solved. The transcriptional activity of the 3'RR has been mentioned previously. Here, we summarize recent findings on the relationship between 3'RR transcriptional activity and its regulatory function.

The zinc finger MYND-type containing 8 (ZMYND8) protein is a histone mark reader that associates with enhancers and promoters and can mediate transcriptional activation or repression in a context-dependent manner (72). ZMYND8 was recently identified as a critical regulator that binds both Eµ and the 3'RR (72). Conditional deletion of the mouse *Zmynd8* gene severely reduced ST and CSR to all isotypes but had no effect on Sµ transcription (72). Significantly, the loss of ZMYND8 led to a substantial increase of RNAPII loading as well as transcription at the 3'RR (notably at hs1,2 and hs3b enhancers) (72).

These findings suggested that ZMYND8-mediated control of the 3'RR function was effected through downregulation of its transcriptional activity, and it was proposed that by suppressing RNAPII loading on the 3'RR, ZMYND8 would suppress competition for transcription factors, thus favoring ST (72).

Another study addressed the role of 3'RR transcription and its eRNAs in the control of ST by using a conditional knockout enabling depletion of the general RNAPII elongation factor SPT5 (136), previously shown to be required for AID recruitment (137). Depletion of SPT5 severely reduced nascent transcription and RNAPII occupancy at downstream S regions but had only a moderate effect at the Sµ region (136). 3C-qPCR assays revealed reduced Eµ/3'RR/Iγ1 interaction frequencies in IL4-activated splenic B cells (136). The apparent decrease of 3'RR transcription in activated SPT5-depleted B cells did not affect its chromatin accessibility or H3K27Ac levels. The depletion also did not significantly impact Mediator and Cohesin recruitment at Eµ, 3'RR, and Iγ1 promoter.

These and other findings suggested that the 3'RR chromatin was in an active state; nonetheless, the weakly transcribed 3'RR was unable to physically interact with its target promoters. This indicated that SPT5-mediated transcription of the 3'RR was required for 3'RR interactions (136). Restoration of transcription through dCas9-VPR at one or two 3'RR enhancers additively rescued 3'RR/Iγ1 promoter interactions and Sγ1 transcription (136). Pharmacological inhibition of transcription initiation or elongation in activated wild-type B cells led to a significant decrease of 3'RR eRNAs. Surprisingly, 3'RR interaction frequencies as assayed by 3C-qPCR assays tended to increase. These findings suggested that transcription elongation within the 3'RR may rather disrupt 3'RR interactions (136).

It was thus proposed that SPT5-mediated transcription of the 3'RR is actually required for the initiation of 3'RR/promoter interactions. Once established, these interactions no longer require 3'RR transcription for their maintenance. Overall, transcription of the 3'RR, but not eRNAs themselves, would be important for 3'RR interactions (136).

#### 4.4.2 The *lncCSR^IgA^* Locus: Controlling the *IgH* Locus From Within May not Be Enough

The eRNA levels are generally lower than the messenger RNA levels of their target genes, which complicates the analysis of the eRNA function(s). Fortunately, a subset of eRNAs are sensitive to the RNA surveillance machinery, the RNA exosome complex, and can therefore be more easily studied in the absence of the RNA exosome (64). In this context, recent analyses of the role of RNA exosome in B cells revealed a novel mechanism that influences CSR, involving long-range interactions between a non-*Ig* locus and the 3'RR. The non-*Ig* locus was termed *lncCSR^IgA^* and is located some 2.6 Mb downstream of the 3'RR (59) (**Figure 2**).

The *lncCSR^IgA^* locus is a divergent eRNA-expressing element which, as detected by 3C assay, interacted with hs4 enhancer of the 3'RR (59). In CH12 cells, deletion of the *lncCSR^IgA^* locus reduced Sα transcription and IgA CSR and decreased the interaction frequency between hs4 enhancer and the deleted locus (59). In *lncCSR^IgA^*-deficient mice, no difference in the distribution of MZ B cells and FO B cells was seen in the spleen. However, activated splenic B cells displayed CSR defect to both IgG2b and IgA, while Peyer’s patch B cells had reduced IgA CSR specifically (138). Based on its DNase I hypersensitivity, MED1 binding, and enrichment in H3K27Ac and H3K4me1 marks, the *lncCSR^IgA^* locus was suggested to act as an enhancer-like element (138).

The *lncCSR^IgA^* is flanked in particular by a CTCF- and Cohesin-binding element, and lies within a TAD that is separated from the *IgH* TAD by other non-*Ig* TADs. The CBE of the *lncCSR^IgA^* locus interacted in particular with the hs4 region of the 3′RR. Accordingly, interaction frequency between hs4 and the CBE dropped following deletion of the *lncCSR^IgA^* locus (138). Various genetic and biochemical analyses pointed toward a pivotal role of the *lncCSR^IgA^* CBE in the intra-TAD^lncCSRIgA^ interactions required for optimal IgA CSR (138). These findings led to a model positing that the transcribed enhancer-like *lncCSR^IgA^* locus produces a lncRNA that facilitates the recruitment of regulatory proteins such as the Cohesin subunit SMC3 to the neighboring CBE. This recruitment alters in turn the interactions that take place within the TAD^lncCSRIgA^ as well as interactions with the 3′RR (138).

The precise mechanism by which CSR is impaired in the absence of the lncCSR^IgA^ RNA remains unclear. Nonetheless, these investigations reveal an unanticipated mechanism whereby the 3'RR-mediated control of CSR within the *IgH* TAD is influenced by chromatin interactions that take place within a different and distant TAD.

### 4.5 Chromatin Loop Extrusion and CSR Center: A Center at Last

The standard loop extrusion model (130, 139–142) stipulates that the ring-shaped cohesin complex binds and passes chromatin through its lumen to form a loop. The process continues until chromatin reaches a CTCF homodimer, at convergent CBEs, which generally blocks loop extrusion (122, 123, 125). In this process, Cohesin not only associates with CBE-bound CTCF but plays an active role within the chromatin loop by promoting for instance enhancer/promoter interactions (122, 123). Additionally, Cohesin may escape the constrains of the CTCF loops, by moving past CTCF anchors, and promote long-range interactions between compartmental domains (122, 123).

Recent studies (61, 143) involved specific transcribed, Cohesin-binding elements in the mechanism that underlie the long-range control of CSR through Cohesin-based impediment of loop extrusion. In one study (61), the V(D)J recombination center (144) of an A-MuLV pro-B line that constitutively transcribes Sγ2b was engineered so that RAG scanning activity (145) was directed toward the *C_H_* region (61). The detected Eµ/Sγ2b/3'CBE interactions were associated with RAD21 binding at the 3'CBEs and a rather low accumulation at Eµ–Sµ and Iγ2b-Sγ2b regions (61). The transcribed Sγ2b region impeded loop extrusion and RAG scanning activity, the latter being specifically detected at the transcribed Sγ2b and the weakly transcribed 3'CBEs. Removal of the active Iγ2b promoter suppressed Sγ2b transcription, RAG scanning, Eµ interactions, and RAD21 accumulation at Sγ2b, but RAG activity now increased at the 3'CBEs (61). These and other findings led to a model stipulating that transcription of the Sγ2b region impedes both upstream and downstream loop extrusions (17, 61).

The other study (143) investigated the mechanism of CSR in splenic B cells and CH12 cells, both in an AID-deficient background. In unstimulated B cells, robust transcription took place at the Eμ region and the 3'RR essentially, and the Eµ region/3'RR/3'CBE interactions formed what was called a CSR center (CSRC) (17, 143, 146). RAD21 and NIPBL were shown to accumulate at the Eµ region, 5'hs1RI, 3'RR, and 3'CBEs (143). Upon stimulation, Eµ/3'RR/3'CBE interactions now included the transcribed switch regions, with a marked accumulation of Cohesin at switch regions (143). This suggests that Cohesin loading at transcribed switch regions contributes to ongoing 3'RR–3'CBE domain extrusion that promotes switch region alignment to initiate CSR (143).

In both unstimulated and stimulated AID-deficient CH12 cells, interactions between the constitutively transcribed Iμ–Cμ, Iα–Cα, 3'RR, and proximal 3'CBE regions were detected, and NIPBL and Cohesin markedly accumulated at the active Iα promoter but not at the other (silent) I promoters (17, 143). Deletion of Iα promoter suppressed the transcription of Sα and IgA CSR and led to a low to moderate increase in upstream S regions' transcription (60, 143). This resulted in the loss of Eμ- and hs4-mediated CSRC interactions with the Sα region. In contrast, interactions of Eμ and hs4 with the newly transcribed sequences upstream of the Sα region were now increased (143).

**Figure 3:**
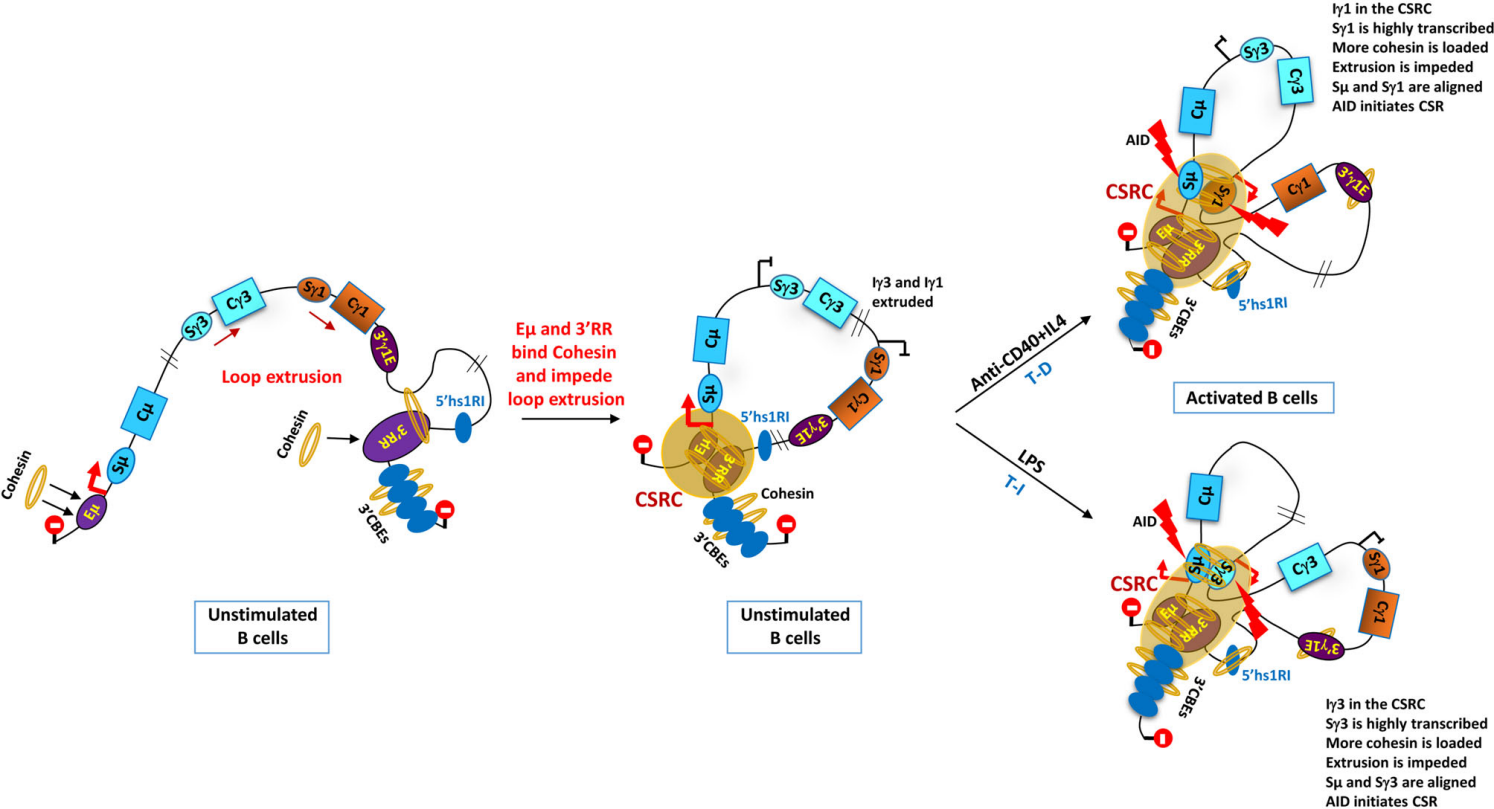
Outline of loop extrusion and class switch recombination center (CSRC) model. The model stipulates that Eµ and 3'RR act as dynamic impediments to loop extrusion thanks to their function as Cohesin-loading sites. The Eµ region impedes upstream extrusion and the 3'RR (potentially assisted by the 3'CBEs) impedes downstream extrusion (illustrated by the stop signals). Chromatin extrusion ultimately leads to a juxtaposition of the Eµ–Sµ region with the 3'RR and 3'CBEs to form a CSRC. Signal-dependent promoters, Iγ1 and Iγ3 (in this example), are primed following anti-CD40+IL4 and LPS stimulations respectively, which mimic T-dependent (T-D) and T-independent (T-I) immune responses, respectively. Ongoing extrusion brings the associated transcribed S regions close to the 3'RR in the CSRC. There, the highly transcribed S regions load more Cohesin and impede chromatin extrusion ultimately aligning the partner S region with Sµ. AID is recruited by the transcribed S regions and initiates bona fide CSR [see (17, 143) for more details]. The 3'γ1E and 5'hs1RI are also Cohesin-loading sites, but their potential role in loop extrusion and CSRC is still unclear [adapted from (17)].

These and other genetic and mechanistic analyses (17, 143, 146) led to a general model positing that Eµ and 3'RR enhancers, as Cohesin-loading sites, act as dynamic impediments to loop extrusion (**Figure 3**).

As discussed (17), the role of Eµ enhancer in this process and its relevance for CSR remain unclear, as ST, CSR, and 3'RR/Sµ region interactions are only marginally affected in its absence (43–45, 67). Moreover, a role for CBEs upstream of Eµ cannot presently be excluded (17). Whether the functions of Eµ as a transcriptional enhancer and as a loop extrusion impediment involve the same mechanisms remains to be elucidated. On the other hand, the 3'γ1E and 5'hs1RI, which control ST of and CSR to specific isotypes (46, 47), are also Cohesin-loading elements. Whether they are involved in loop extrusion impediment is still unclear. Thus, the mechanisms that regulate loop extrusion during CSR remain to be investigated and more so because not all loops are Cohesin-dependent (147).

In this context, a recent study involved the RNA exosome complex in the regulation of chromatin loop extrusion. By generating a conditional mutant mouse line to induce loss of the DIS3 RNase subunit of the RNA exosome complex, it was shown in particular that this loss led to decreased binding of CTCF and Cohesin (RAD21) at the 3'CBEs and 5'hs1RI, which was often associated with accumulated eRNAs (148). Interestingly, this overlap between reduced CTCF/Cohesin occupancy and accumulated eRNAs correlated with an accumulation of DNA/RNA hybrids at the 3'CBEs, 3'RR, and switch regions, particularly at the Sµ region. These findings, together with the observation that 3'RR/Eµ interactions were reduced upon loss of DIS3 activity, suggested that the accumulation of DNA/RNA hybrids at specific transcribed sequences impeded Cohesin-mediated chromatin loop extrusion during CSR (148).

Thus, by processing non-coding RNAs at critical transcribed sequences, the RNA exosome complex emerges as an important factor in the mechanisms that regulate chromatin loop extrusion.

## 5 Perspectives

The last decade witnessed important advances in our understanding of the transcriptional and epigenetic mechanisms involved in the long-range control of CSR. Elucidation of the function of newly identified regulatory elements and the role of *trans*-acting factors in CSR added new layers to the complexity of the mechanisms involved. The development of various genome editing approaches as illustrated by CRISPR/Cas9-based techniques as well as high-throughput technologies made it possible to tackle and to further our knowledge of the long-range chromatin interactions that take place during CSR.

As usual, any new knowledge raises new questions and paths. For instance, the question of why do some long-range regulatory elements target specific promoters remains to be investigated. The signals that trigger chromatin loop formation and their collapse and the precise relationship between (presumably) large chromatin loops and the fine details of transcriptional and epigenetic control are still unclear. In the context of Cohesin-based loop extrusion/CSRC model, the role of other transcriptional/architectural factors remains to be investigated. Moreover, one should bear in mind that *IgH* chromatin domains are defined in resting or activated B-cell populations and, therefore, display averaged interactions [e.g., (149)] that do not necessarily reflect interactions on a single-cell or single-chromosome basis. Correlatively, it is presently unclear to what extent the long-range mechanisms identified in *in vitro*-activated B-cell populations operate during genuine T-cell-dependent and T-cell-independent responses. Though technically challenging, it will be of outmost importance to develop new approaches and models to tackle these mechanisms on a single B-cell or chromosome basis during immune responses.

## Author Contributions

AK wrote the manuscript. AD contributed to the writing of the manuscript and checked the references and figures. All authors contributed to the article and approved the submitted version.

## Funding

This work was supported by the Agence Nationale de la Recherche (ANR-16-CE12-0017), the Institut National du Cancer (INCA_9363, PLBIO15-134), the Fondation ARC pour la Recherche sur le Cancer (PJA 20191209515), and the Ligue Contre le Cancer (Ligue Régionale: comités de l’Ex Région Midi-Pyrénées).

## Conflict of Interest

The authors declare that the research was conducted in the absence of any commercial or financial relationships that could be construed as a potential conflict of interest.

## Publisher’s Note

All claims expressed in this article are solely those of the authors and do not necessarily represent those of their affiliated organizations, or those of the publisher, the editors and the reviewers. Any product that may be evaluated in this article, or claim that may be made by its manufacturer, is not guaranteed or endorsed by the publisher.
